# Regulation of lipid droplet size and phospholipid composition by stearoyl-CoA desaturase[S]

**DOI:** 10.1194/jlr.M039669

**Published:** 2013-09

**Authors:** Xun Shi, Juan Li, Xiaoju Zou, Joel Greggain, Steven V. Rødkær, Nils J. Færgeman, Bin Liang, Jennifer L. Watts

**Affiliations:** * School of Molecular Biosciences, Washington State University, Pullman, WA 99164-7520; † Key Laboratory of Animal Models and Human Disease Mechanisms, Kunming Institute of Zoology, the Chinese Academy of Sciences, Kunming, Yunnan 650223, China; § School of Life Science, University of Science and Technology of China, Hefei 230036, China; ** Department of Life Sciences and Biotechnology, Kunming University, Kunming, Yunnan 650214, China; and; †† Department of Biochemistry and Molecular Biology, University of Southern Denmark, 5230 Odense M, Denmark

**Keywords:** fatty acid synthesis, *Caenorahbditis elegans*, phosphatidylcholine, phosphatidylethanolamine, fatty acid oxidation, oleic acid

## Abstract

Fatty acid desaturation regulates membrane function and fat storage in animals. To determine the contribution of stearoyl-CoA desaturase (SCD) activity on fat storage and development in the nematode *Caenorhabditis elegans*, we analyzed the lipid composition and lipid droplet size in the *fat-6;fat-7* desaturase mutants independently and in combination with mutants disrupted in conserved lipid metabolic pathways. *C. elegans* with impaired SCD activity displayed both reduced fat stores and decreased lipid droplet size. Mutants in the *daf-2* (insulin-like growth factor receptor), *rsks-1* (homolog of p70S6kinase, an effector of the target of rapamycin signaling pathway), and *daf-7* (transforming growth factor β) displayed high fat stores, the opposite of the low fat observed in the *fat-6;fat-7* desaturase mutants. The metabolic mutants in combination with *fat-6;fat-7* displayed low fat stores, with the exception of the *daf-2;fat-6;fat-7* triple mutants, which had increased de novo fatty acid synthesis and wild-type levels of fat stores. Notably, SCD activity is required for the formation of large-sized lipid droplets in all mutant backgrounds, as well as for normal ratios of phosphatidylcholine (PC) to phosphatidylethanolamine (PE). These studies reveal previously uncharacterized roles for SCD in the regulation of lipid droplet size and membrane phospholipid composition.

During the past 50 years, humans world-wide have increased their caloric intake beyond what is required for body mass maintenance. Consequently, the rates of obesity, metabolic syndrome, and type 2 diabetes have steadily increased. Stearoyl-CoA desaturase (SCD), also known as Δ9 desaturase, is a key enzyme in the de novo lipogenic pathway. SCD1 deficiency in mice leads to decreased fat stores and increased fat oxidation; consequently, deficient mice are resistant to diet-induced obesity and are protected from lipotoxicity induced by saturated fats (1, 2). Human studies have identified genetic variations in human SCD1 associated with body fat distribution, insulin sensitivity, and metabolic syndrome (3, 4).

SCD is responsible for the formation of monounsaturated fatty acids from saturated fatty acids by catalyzing the insertion of a double bond into the ninth carbon of saturated C16 and C18 substrates. Monounsaturated fatty acids are preferred substrates for the synthesis of triacylglycerol (TAG), as well as for membrane phospholipids and sphingolipids (5). The *C. elegans fat-5;fat-6;fat-7* triple-mutant strain, which is completely deficient in SCD activity, is lethal, but the *fat-6;fat-7* double mutants, similar to their mouse SCD1 counterparts, are viable but have decreased fat stores and increased expression of fat oxidation genes (6, 7). The *fat-6;fat-7* strain also exhibits slow growth, reduced brood size, cold sensitivity, and greatly altered fatty acid composition (7). Unlike most animals, *C. elegans* contains a Δ-12 fatty acid desaturase (FAT-2) that enables it to synthesize polyunsaturated fatty acids from oleic acid (8). Polyunsaturated fatty acids (PUFA) are not present in the *C. elegans* laboratory diet, because they are not synthesized by *E. coli*. Thus, in *C. elegans*, SCD is the first desaturase required for the biosynthesis of a wide range of PUFAs (9).

Lipid synthesis and oxidation are regulated by various nutrient and energy sensing pathways. These include the conserved insulin/insulin growth factor-1 (IIS) signaling pathway, the target of rapamycin (TOR) pathway, the transforming growth factor β (TGFβ) pathway, and the AMP-activated protein kinase pathway (AMPK). *C. elegans* mutants with deficient IIS or TGFβ signaling, such as *daf-2* and *daf-7*, tend to arrest during development as dauer larvae, and under conditions where the mutants reach adulthood, they are resistant to environmental stresses, have a greatly extended lifespan, and store excess fat compared with wild-type (WT) (10–14). Similarly, the *C. elegans* TOR mutant *let-363*, along with Raptor mutant *daf-15*, arrest in larval stages with excess fat stores (15–17). Mutants in the gene encoding the TOR complex 2 component Rictor, *rict-1*, do not arrest as larvae, but they exhibit slow growth, increased fat stores, and shortened lifespan (18, 19). Similarly, *rsks-1* mutants in the downstream ribosomal S6 kinase also exhibit slow growth and altered lifespan (20). Finally, overexpression of the *C. elegans* AMPK gene *aak-2* increases lifespan (21), and AAK-2 activity is necessary for proper energy expenditure during dauer larval stages (22).

Lipid droplets are fat-storing organelles consisting of a hydrophobic core of TAG and cholesterol ester surrounded by a phospholipid monolayer containing various proteins (23). Proteomic studies of lipid droplets from various organisms, including *C. elegans,* reveal that lipid droplets are associated with a complex mixture of proteins that are predicted to play roles in lipid synthesis and degradation, membrane trafficking, and protein degradation (24, 25). *C. elegans* contains lipid droplets in intestinal, hypodermal, and gonadal tissues (9, 26).

The *fat-6;fat-7* strain exhibits opposite fat storage phenotypes compared with the *daf-*2 (IIS), *daf-7* (TGFβ), and previously characterized mutants in TOR signaling. The SCD-deficient *fat-6;fat-7* double mutants have reduced fat stores, whereas the other mutants cause nematodes to store higher amounts of TAG. We constructed triple-mutant strains to determine whether SCD activity is necessary for high fat stores in these strains. We found a striking requirement for endogenous SCD activity for the regulation of lipid droplet size and discovered that SCD activity influences the relative ratios of membrane phospholipid species.

## MATERIALS AND METHODS

### Nematode strains and growth conditions

Nematode growth media (NGM) was used to maintain *C. elegans* with the *E. coli* (OP50) at 20°C. The WT strain was N2. The strains used in this study were BX106 *fat-6(tm331)*, BX107 *fat-5(tm420)*, BX110 *fat-5(tm420);fat-6(tm331)*, BX156 *fat-6(tm331);fat-7(wa36)*, CB1370 *daf-2(e1370)*, CB1372 *daf-7(e1372)*, RB754 *aak-2(ok524)*, KQ6 *rict-1(mg360)*, RB1206 *rsks-1(ok1255)*, and HA1947 *sams-1-(ok3033)*. The RB strains were outcrossed four times to N2. Double- and triple-mutant strains constructed for this study were BX168 *daf-2(e1370);fat-6(tm331)*, BX250 *daf-2(e1370);fat-5(tm420)*, BX251 *daf-2(e1370);fat-5(tm420);fat-6(tm331)*, BX177 *daf-2(e1370);fat-6(tm331);fat-7(wa36)*, BX217 *aak-2(ok524);fat-6(tm331);fat-7(wa36)*, and BX218 *rsks-1(ok1255);fat-6(tm331);fat-7(wa36).* Fatty acid supplementation was achieved by adding sodium oleate (NuChek Prep) at a final concentration range of 0.1–0.5 mM to NGM media containing 0.1% tergitol (NP40). Fatty acid stock solutions were added after autoclaved media cooled to 50°C. Feeding RNAi was performed on NGM plates supplemented with 100 μg/ml ampicillin and 2 mM isopropyl-β-D-thiogalactopyranoside (ITPG) and *E. coli* strain HT115 (27).

### Fatty acid composition and lipid analysis

Fatty acid composition of young adult nematodes was determined by gas chromatography/mass spectrometry (GC/MS) as previously described (6, 28). Separation of the TAG and phospholipid fractions used a two-solvent TLC protocol. Approximately 10,000 young adult stage *C. elegans* were washed from NGM plates several times in water. Most of the water was removed, and worm pellets were frozen in liquid nitrogen. Lipids were extracted by adding 5 ml of ice-cold chloroform:methanol (1:1) and incubating overnight at −20° with occasional shaking. A solution of 0.2M H_3_PO_4_ and 1M KCl was added to samples, which resulted in phase separation of the organic and aqueous phase. The organic phase was removed and dried under argon, then resuspended in chloroform. Samples were loaded in triplicate, and TLC plates were developed two thirds of the way up the plate in the first solvent system: chloroform:methanol:water:acetic acid (65:43:3:2.5), dried, and then the second solvent system hexane:diethylether:acetic acid (80:20:2) was developed to the top of the plate. Lipids were visualized under UV light after spraying the plate with 0.005% primuline, and spots corresponding to TAG and the major phospholipids were scraped, spiked with a known standard (15:0), and transesterified for GC/MS analysis to determine the fatty acid composition as well as to determine the relative levels of TAG and phospholipid (PL) fractions. At least three biological replicates were used for TLC analysis. Significance was determined with one-way ANOVA analysis and Tukey's multiple comparison posttest using GraphPad Prism 5 software.

Stable isotope labeling of fatty acids was performed essentially as described (29). Briefly, equal amounts of bacteria grown in either LB (^12^C media) or isogrow (98.5% ^13^C-enriched, Sigma) were mixed and plated onto agarose plates. For each sample, approximately 30,000 synchronized L1 nematodes prepared from hypochlorite treatment of gravid adults were added to the plates and grown for 48 h at 20°C (worms reached L4 larval stage). Nematodes were washed off the plates, their lipids were extracted, and fatty acids were analyzed by GC/MS as described (29). Isotopomers were monitored in a scanning ion mode corresponding to the fatty acid species of interest: 16:0 was scanned from *m/z* 270–286, 18:0 was scanned from *m/z* 298–316, and 18:1(n-7) was scanned from *m/z* 296–314.

#### Quantification of lipid droplet size.

For measurement of lipid droplet size, at least ten young adult worms of each genotype stained with postfixed Nile Red were photographed (13). For each worm photograph, a 26 × 26 μm square was placed arbitrarily over the mid-intestinal region, and within the square, each visible Nile Red-stained droplet was manually traced using the circle tool of Image Pro Plus software, which recorded the diameter of each droplet. For each worm, the average lipid droplet diameter was calculated. Statistical comparisons (one-way ANOVA and Tukey's multiple comparison test) were performed using GraphPad Prism 5 software.

### Fatty acid oxidation assay

Fatty acid oxidation was performed on L4 nematodes essentially as described (30), except that tritiated palmitic acid (30 Ci/mmol, Perkin Elmer, Waltham, MA) was used as substrate.

### Quantitative RT-PCR analysis

WT, *fat-6;fat-7*, and *daf-2;fat-6;fat-7* nematodes were synchronized and harvested at L4 stage. RNA and cDNA was prepared as described (7). Real-time PCR assays were run and monitored with an ABI Prism 7000 Sequence Detection system (Applied Biosystems, Foster City, CA). The real-time PCR was conducted on three treatment groups, with each individual treatment group in triplicate. Threshold values (Ct) for the gene of interest and a housekeeping gene *tbb-2* were determined using ABI Prism SDS software version 1.1 (Applied Biosystems). The expression level of the gene of interest was evaluated using the 2^−(ΔΔCt)^ method (31).

### Physiological assays

#### Growth rate.

Eggs were isolated from gravid adults using hypochlorite treatment and then plated onto NGM plates seeded with *E. coli* strain OP50. The number of adults and total number of nematodes were determined at various time points.

#### Brood size.

For analysis of total progeny produced per worm, 10–20 L4s were transferred individually to fresh NGM plates seeded with *E. coli* strain OP50. Worms were transferred daily until they did not produce any more progeny. Two days after removal of the adult, the live progeny of each genotype were counted.

## RESULTS

### SCD activity is necessary for large-sized lipid droplets

**Fig. 1.**
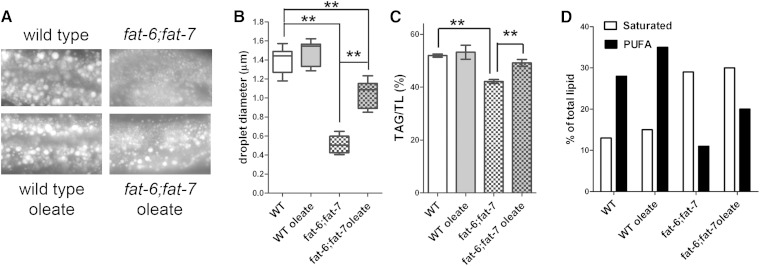
SCD activity is required for large lipid droplets and normal lipid composition. (A) The size of lipid droplets in the mid-intestinal region of young adult *C. elegans* depends on SCD. Young adults were fixed and stained with Nile Red to visualize lipid droplets. Fluorescent images of lipid droplets in the mid-intestine show a range of lipid droplet diameters in WT and *fat-6;fat-7*, unsupplemented and supplemented with 0.2 mM oleate. (B) Quantification of lipid droplet diameter in the mid-intestinal region. (C) Quantitative analysis of TAG levels demonstrates that SCD is required for normal TAG levels and that 0.2 mM dietary oleate increases TAG levels in SCD-deficient *C. elegans*. (D) Simplified fatty acid composition of WT and *fat-6;fat-7* strains, unsupplemented or supplemented with 0.2 mM sodium oleate. Complete fatty acid composition values with standard errors are reported in supplementary Table I. Error bars are SEM, **P < 0.01.

In the nematode *C. elegans*, the lack of SCD activity in the *fat6;fat-7* double-mutant strain has profound consequences for the fatty acid composition of membrane phospholipids and TAG storage lipids (7). In our previous study, we showed faint Nile Red staining in *fat-6;fat-7* animals, which increased in intensity when the animals were supplemented with 0.1 mM sodium oleate (7). However, recent work in many labs has demonstrated that Nile Red fluorescence in live animals does not correlate with actual TAG stores but instead stains lysosome-related organelles (13, 32–34). In contrast, the intensity of Nile Red, Sudan black, or Oil-Red O staining in nematodes that have been fixed with isopropanol or paraformaldehyde corresponds well with biochemical measurements of TAG (13, 33) and stains lipid droplets, not lysosome-related organelles (35). We therefore analyzed the lipid-staining pattern of fixed, young-adult-stage nematodes (with 0–2 eggs), and we found that the SCD-deficient *fat-6;fat-7* double mutants have fewer and smaller lipids droplets than WT controls (**Fig. 1A**). We used imaging software to measure lipid droplet diameter of cross-sections of the mid-intestinal region to determine the frequency of lipid droplets of various sizes. The individual lipid droplets in WT ranged in diameter from 0.5 μm to 3.7 μm, with an average size of 1.4 μm, while lipid droplets in *fat-6;fat-7* were much smaller, with a range from 0.2 μm to 1.5 μm, and an average diameter of 0.5 μm (Fig. 1B). In addition, we examined lipid droplet sizes from the single SCD mutant strains *fat-5*, *fat-6*, and *fat-7* single mutants, as well as the *fat-5;fat-6* double mutant, and we found that the lipid droplet diameters in these strains do not appreciably differ from WT (supplementary Fig. I-A). Thus, the *C. elega*ns SCD strain with the most severe lipid droplet-size defect is *fat-6;fat-7*, which also has the most severe fatty acid composition defect among the SCD single- and double-mutant strains (6, 7).

Next, we analyzed the fatty acid composition of individual membrane phospholipids in the *fat-6;fat-7* double mutants. In WT, PUFA is detected mostly in the phospholipid fraction, in contrast to TAG, which contains only small amounts of PUFA (7, 29, 36). When SCD activity is blocked by simultaneous mutations in *fat-6* and *fat-7*, normal PUFAs are not formed, because the FAT-6 and FAT-7 desaturases are required to synthesize PUFA de novo from acetyl CoA or to synthesize them from fatty acid precursors (primarily palmitic acid) derived from the bacterial diet (7, 29). In the absence of FAT-6 and FAT-7, unusual C18 PUFAs are synthesized via desaturation and elongation of palmitoleic acid (16:1n-7), which is synthesized from palmitic acid by the Δ9 desaturase FAT-5 (7, 29). For this study, we examined the fatty acid composition of TAG faction, as well as the membrane phospholipids phosphatidylcholine (PC), phosphatidylethanolamine (PE), phosphatidylserine (PS), and phosphatidylinositol (PI) in the *fat-6;fat-7* double mutants. Similar to the distribution of PUFAs in WT lipids, we detected the unusual C18 PUFAs in PI and PS, as well as PC, but they are present to a lesser extent in PE and in the TAG fraction (supplementary Table I). In agreement with our previous studies, *fat-6;fat-7* had overall lower levels of TAG than WT (Fig. 1C, **Table 1**) and a greatly increased composition of saturated fatty acids and decreased composition of polyunsaturated fatty acids (Fig. 1D).

**TABLE 1.** Relative percentage of phospholipid classes and TAG in WT (N2) and *fat-6;fat-7* double mutants, grown without fatty acids and supplemented with 0.2 mM sodium oleate (18:1n-9)

	Relative % of Phospholipids					
	PC	PE	PI	PS	PC:PE	% TAG/TL
WT	54.1% (1.2)^a^	35.7% (0.9)^a^	5.1% (0.6)^a^	5.1% (0.1)^a^	1.51	52.0% (0.6)^ad^
WT 0.2 mM oleate	53.5% (2.3)^a^	34.4% (2.0)^a^	6.4% (0.2)^a^	5.8% (0.2)^a^	1.55	53.2% (2.7)^a^
*fat-6;fat-7*	45.5% (0.8)^b^	40.1% (0.1)^b^	5.6% (0.2)^a^	7.8% (0.6)^a^	1.13	42.2% (0.7)^b^
*fat-6;fat-7* oleate	44.1% (0.2)^b^	42.4% (0.9)^b^	5.7% (0.3)^a^	7.1% (1.6)^a^	1.04	48.8% (1.2)^cd^

Values are the average (SEM) from three independent lipid extractions of young adult stage *C. elegans*. Those not sharing a common letter within the same column differ (*P* < 0.05)

### Dietary oleic acid promotes PUFA production but does not restore lipid droplet size to WT levels

Our previous work showed that the slow growth and reduced brood size in *fat-6;fat-7* worms were partially rescued by dietary oleic acid (7). To see whether dietary oleic acid can rescue the lipid droplet and fat storage defects of *fat-6;fat-7* we grew the worms on plates containing 0.1–0.5 mM sodium oleate. We examined the lipid droplet size in the oleate-supplemented worms and found they had a larger size distribution compared with unsupplemented *fat-6;fat-7* worms, indicating that dietary oleate induces large-sized lipid droplets in SCD-deficient worms, although oleate levels up to 0.5 mM cannot fully rescue lipid droplet size to that of WT (Fig. 1A, B, supplementary Fig. I-B). GC/MS analysis revealed that fatty acid uptake did not increase significantly above that seen in 0.2 mM oleate; therefore, the 0.2 mM concentration was chosen for detailed lipid analysis. Analysis of TAG and phospholipid fractions indicated that 0.2 mM dietary oleate also significantly increased the TAG levels of the *fat-6;fat-7* strain (Fig. 1C, Table 1). The 0.2 mM concentration of oleate resulted in an incorporation of oleic acid and its downstream products [linoleic acid (18:2n-6), γ-linolenic acid (18:3n-6), dihommogamma-linolenic acid (20:3n-6), and eicosapentaenoic acid (20:5n-3)] such that they made up 15–17% of total fatty acids in the supplemented *fat-6;fat-7* worms (supplementary Table I). Interestingly, the fatty acid composition of the supplemented animals still showed high levels of stearic acid (18:0), similar to unsupplemented *fat-6;fat-7* double mutants, and significant amounts of unusual C18 PUFAs (6%, compared with 12% in unsupplemented double mutants and undetectable in WT animals). Thus, dietary oleate promoted PUFA synthesis in both WT and *fat-6;fat-7*, and it altered the fatty acid composition profile of *fat-6;fat-7* animals but did not restore it to WT (Fig. 1D, supplementary Table I). The failure of dietary oleate to completely rescue lipid droplet size in *fat-6;fat-7* worms indicates a requirement for endogenous synthesis of unsaturated fatty acids for optimal lipid droplet size and TAG stores.

### Interactions between SCD and conserved metabolic pathways regulating fat storage and fat oxidation

To examine the role of SCD in the formation of lipid droplets, TAG storage, and growth and development, we generated the triple-mutant strains that allowed us to examine the role of SCD activity in the background of mutations that confer high fat stores or altered fat oxidation.

#### AMPK mutants have WT lipid composition and do not suppress low fat in SCD-deficient worms.

**Fig. 2.**
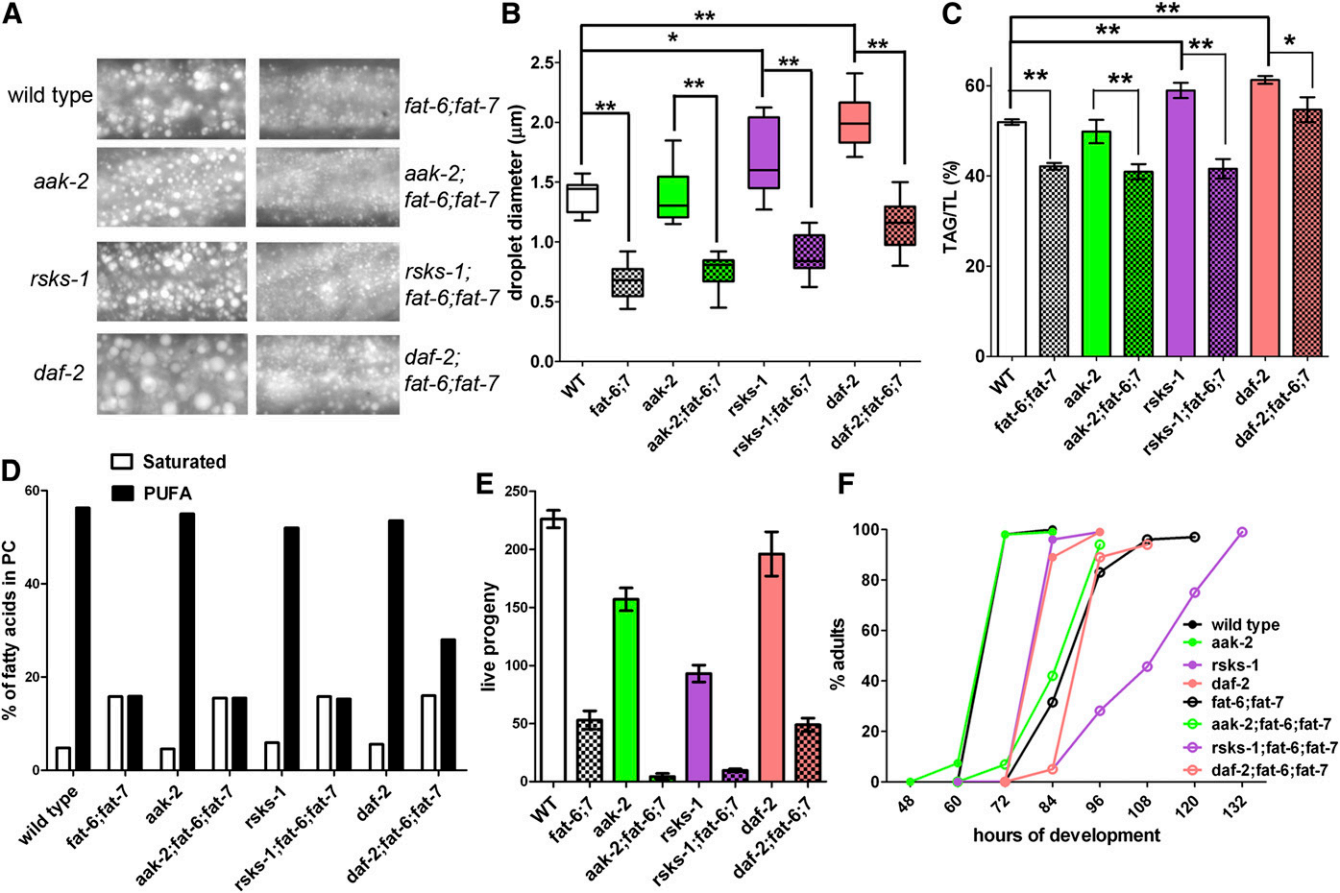
SCD activity is required for large-sized lipid droplets in high-fat mutants. (A) Large-sized lipid droplets in *daf-2* and *rsks-1* mutants require SCD. Young adults were fixed and stained with Nile Red to visualize lipid droplets in the mid intestine. (B) Quantification of lipid droplet diameters in the mid-intestinal region. (C) Quantitative analysis of TAG levels demonstrates that SCD is required for TAG stores in all mutant backgrounds except for *daf-2*. (D) Simplified fatty acid composition of WT and mutant strains. Complete fatty acid composition values with standard errors are reported in supplementary Table II. (E) Quantification of brood size of WT and mutant strains. (F) Growth rates of WT and mutant strains. Graph shows the percentage of a population that reached adulthood by the indicated time (0 h = 1–32 cell stage embryo). Error bars are SEM, **P < 0.01, *P < 0.05.

Mutations in the *C. elegans aak-2* gene, which encodes the α subunit of AMPK, affect the regulation of fat oxidation, especially under conditions of starvation (22). Because experiments in *C. elegans* indicate that the low fat stores in SCD mutants are associated with increased expression of fat oxidation genes (7) and because experiments in mice indicate that SCD inhibition leads to activation of AMPK (37), we tested whether the low fat stores in the *fat-6;fat-7* double mutants could be suppressed by a mutation in *aak-2*. We first examined the lipid droplet size and measured the lipid composition of *aak-2* mutants. We found that, under growth conditions providing ample food, the *aak-2* mutants were indistinguishable from WT with respect to lipid droplet size, lipid composition, and growth rate (**Fig. 2A–E**, **Table 2**, supplementary Table II). In addition, the triple-mutant strain *aak-2;fat-6;fat-7* was indistinguishable from *fat-6;fat-7* with respect to lipid droplet size, lipid composition, and growth rate (Fig. 2A–E, Table 2, supplementary Table II). We detected a smaller brood size in the *aak-2* mutant compared with WT and a severely small brood size in the *aak-2;fat-6;fat-7* triple mutant, suggesting a role for AAK-2 in optimal fecundity (Fig. 2F). The severe reduction in live progeny in the triple mutant is consistent with additive developmental defects in the *aak-2* and *fat-6;fat-7* strains.

**TABLE 2.** Relative percentage of lipid classes in WT (N2), *aak-2*, *rsks-1*, *daf-2*, *fat-6;fat-7*, *aak-2;fat-6;fat-7*, *rsks-1;fat-6;fat-7*, and *daf-2;fat-6;fat-7*

	Relative % of Phospholipids					
	PC/PL	PE/PL	PI/PL	PS/PL	PC:PE	% TAG/TL
N2	54.1% (1.2)*^a^*	35.7% (0.9)*^a^*	5.1% (0.6)*^a^*	5.1% (0.1)*^a^*	1.51	52.0% (0.6)*^a^*
*aak-2*	56.4% (1.0)*^a^*	31.7% (1.6)*^a^*	5.7% (0.5)*^a^*	6.2% (0.3)*^a^*	1.78	49.9% (2.6)*^a^*
*rsks-1*	59.6% (2.6)*^a^*	30.4% (4.6)*^a^*	5.4% (0.3)*^a^*	5.1% (1.2)*^a^*	1.92	59.0% (1.7)*^b^**^d^*
*daf-2*	55.8% (1.0)*^a^*	33.0% (1.1)*^a^*	5.0% (0.6)*^a^*	6.1% (0.7)*^a^*	1.69	61.3 (0.9)*^b^*
*fat-6;fat-7*	45.5% (0.8)*^b^*	40.1% (0.1)*^b^*	5.6% (0.2)*^a^*	7.8% (0.6)*^a^*	1.13	42.2% (0.7)*^c^*
*aak-2;fat-6;fat-7*	47.3% (2.4)*^b^*	43.8% (1.4)*^b^*	4.1% (1.5)*^a^*	5.9% (0.9)*^a^*	1.08	41.0% (1.7)*^c^*
*rsks-1;fat-6;fat-7*	45.5% (0.3)*^b^*	43.7% (1.7)*^b^*	4.2% (0.5)*^a^*	6.6% (1.7)*^a^*	1.04	42.8% (3.0)*^c^*
*daf-2;fat-6;fat-7*	46.2% (1.2)*^b^*	40.7% (0.9)*^b^*	6.3% (0.5)*^a^*	6.9% (0.5)*^a^*	1.13	54.7% (2.8)*^a^**^d^*

Values are the average (SEM) of three independent lipid extractions of young adult stage *C. elegans*.

a–d Values not sharing a common letter within the same column differ (*P* < 0.05).

A second isoform of the α subunit of AMP kinase is encoded by the *aak-1* gene. To ensure that AMPK activity was efficiently knocked down, we treated the WT, *aak-2*, and *aak-2;fat-6;fat-7* strains with *aak-1* RNAi. We found no change in lipid droplet size in any of the strains with *aak-1* RNAi treatment (supplementary Fig. II-A). With respect to the regulation of fat storage, our results show no role for AMPK in modulating the fat stores in the SCD mutants.

#### S6K regulates fatty acid composition and fat stores.

TOR is a conserved serine/threonine kinase that regulates growth in response to nutritional signals. Fat content in *C. elegans* is influenced by the TOR pathway, and mutants in conserved members of TOR complexes 1 and 2 show alterations in fat storage (15, 16, 18, 19). In mammals, a downstream target of TOR signaling is the p70 ribosomal S6 kinase, which in *C. elegans* promotes germline proliferation and is required for the proper regulation of lifespan (20, 38). We examined lipid droplet size and fatty acid composition in the TOR complex 2 component *rict-1* mutants as well as in *rsks-1* mutants. We found that, in both strains, the formation of large lipid droplets depends on FAT-6 and FAT-7 (Fig. 2A, B, supplementary Fig. II-B). In contrast, the requirements for RICT-1 and RSKS-1 for producing optimal growth rate and brood size act in parallel to SCD, because the triple mutants have a more severe growth and brood size reduction than the *fat-6;fat-7* strain (Fig. 2E, F, supplementary Fig. II-C).

Because fat composition had not been previously studied in the *rsks-1* mutant, we examined the lipid composition in this mutant strain, as well as in the *rsks-1;fat-6;fat-7* triple-mutant strain. We discovered that *rsks-1* mutants, similar to other mutants in the TOR pathway, have increased TAG content compared with WT (Fig. 2C, supplementary Table II). However, in combination with SCD deficiency, TAG accumulation was similar to the *fat-6;fat-7* double mutant, indicating that SCD activity is required for the accumulation of high fat stores and large lipid droplets in the *rsks-1* mutants (Fig. 2A–D, Table 2, supplementary Table II). Interestingly, while *rict-1* mutants have a nearly WT fatty acid composition (supplementary Fig II-D), all of the lipids in the *rsks-1* strain showed an increase in C20 omega-6 PUFAs (20:3n-6 and 20:4n-6) and a decrease in the C20 omega-3 fatty acids (20:4n-3 and 20:5n-3) (supplementary Fig. II-E, supplementary Table II). We found a fatty acid composition defect similar to *rsks-1* in RNAi treatment of more than 30 ribosomal proteins that we screened [C20 fatty acid composition of *rps-9(RNAi)* is shown in supplementary Fig. II-F]. This indicates that for fatty acid composition, *rsks-1* mutants, not *rict-1*, phenocopy the altered omega-6/omega-3 fatty acid composition changes that result from the disruption of protein translation.

#### Overlapping developmental requirements of DAF-7 and SCD.

The *C. elegans* TGFβ mutant *daf-7*, similar to *daf-2* mutants, tends to arrest as dauer larvae (39). However, when grown under conditions that allow the worms to develop to adulthood, the *daf-7* mutants store excess fat (13, 40). Our attempts to construct the *daf-7;fat-6;fat-7* triple-mutant strain were unsuccessful. We observed that the *daf-7;fat-6* double mutants showed increased incidence of dauer formation at 20°C compared with the *daf-7* strain (data not shown), and after numerous attempts to obtain the triple mutant, only one worm was confirmed by PCR to possess the *daf-7;fat-6;fat-7* genotype, and this nematode did not produce live progeny. Therefore, we conclude that the additive developmental defects of TGFβ and SCD deficiency led to lethality of the *daf-7;fat-6;fat-7* triple mutants.

#### Mutation in daf-2 (IIS) partially suppresses the low TAG content in SCD-deficient worms.

Like the *daf-7* strain, the *daf-2(1370)* mutants, carrying a hypomorphic allele of the IGF receptor, store high amounts of fat (41). We visualized fat stores in WT, *daf-2*, and *daf-2;fat-6;fat-7* triple-mutant strains using Nile Red staining of fixed nematodes, and we found that lipid droplets were significantly larger in the *daf-2* strain than in WT (Fig. 2A). We found that lipid droplets in in *daf-2* mutants ranged 0.5–7.5 μm, with an average size of 2.0 μm (Fig. 2A, B), and, as previously reported (13, 42), the *daf-2* mutant strain contain high levels of TAG and slightly reduced levels of PUFAs (Fig. 2C, D, Table 2, supplementary Table II). The *daf-2;fat-6;fat-7* strain had lipid droplets that ranged 0.3–3.0 μm, with an average lipid droplet size of 1.1 μm, which was closer to the size of the *fat-6;fat-7* strain than was *daf-2* and which was smaller than WT (Fig. 2A, B). This indicates that SCD activity is necessary to produce the large-sized lipid droplets (>3 μm) that are found in WT and *daf-2* mutants. However, qualitative staining with Nile Red indicated that the lipid droplets in *daf-2*, *fat-6;fat-7* worms were more abundant than those in the *fat-6;fat-7* strain, and lipid analysis revealed that the percentage of fatty acids found in the TAG fraction was significantly higher in young adult *daf-2*,*fat-6;fat-7* worms than in *fat-6;fat-7*, similar to the levels measured in similar-aged WT nematodes (Fig. 2C, Table 2). This indicates that, whereas FAT-6 and FAT-7 SCD activity is required to produce large-sized lipid droplets, it is only partially required for increased fat stores in the *daf-2* mutant background. While the *daf-2;fat-6;fat-7* strain contained increased PUFA in all of the lipid classes compared with the *fat-6;fat-7* strain (Fig. 2D, supplementary Table II), the increased fat stores and increased PUFAs did not confer any suppression of the slow growth or reduced brood size of the *fat-6;fat-7* double mutants; in fact, the *daf-2;fat-6;fat-7* triple mutants grew at a slower rate than the *fat-6;fat-7* double mutants (Fig. 2E, F). Taken together, these findings reveal an interaction between IGF signaling and SCD activity in the regulation of fat stores.

### Increased fat stores in *daf-2;fat-6;fat-7* mutants are not a consequence of reduced fat oxidation but are associated with increased de novo fatty acid synthesis and increased FAT-5 activity

**Fig. 3.**
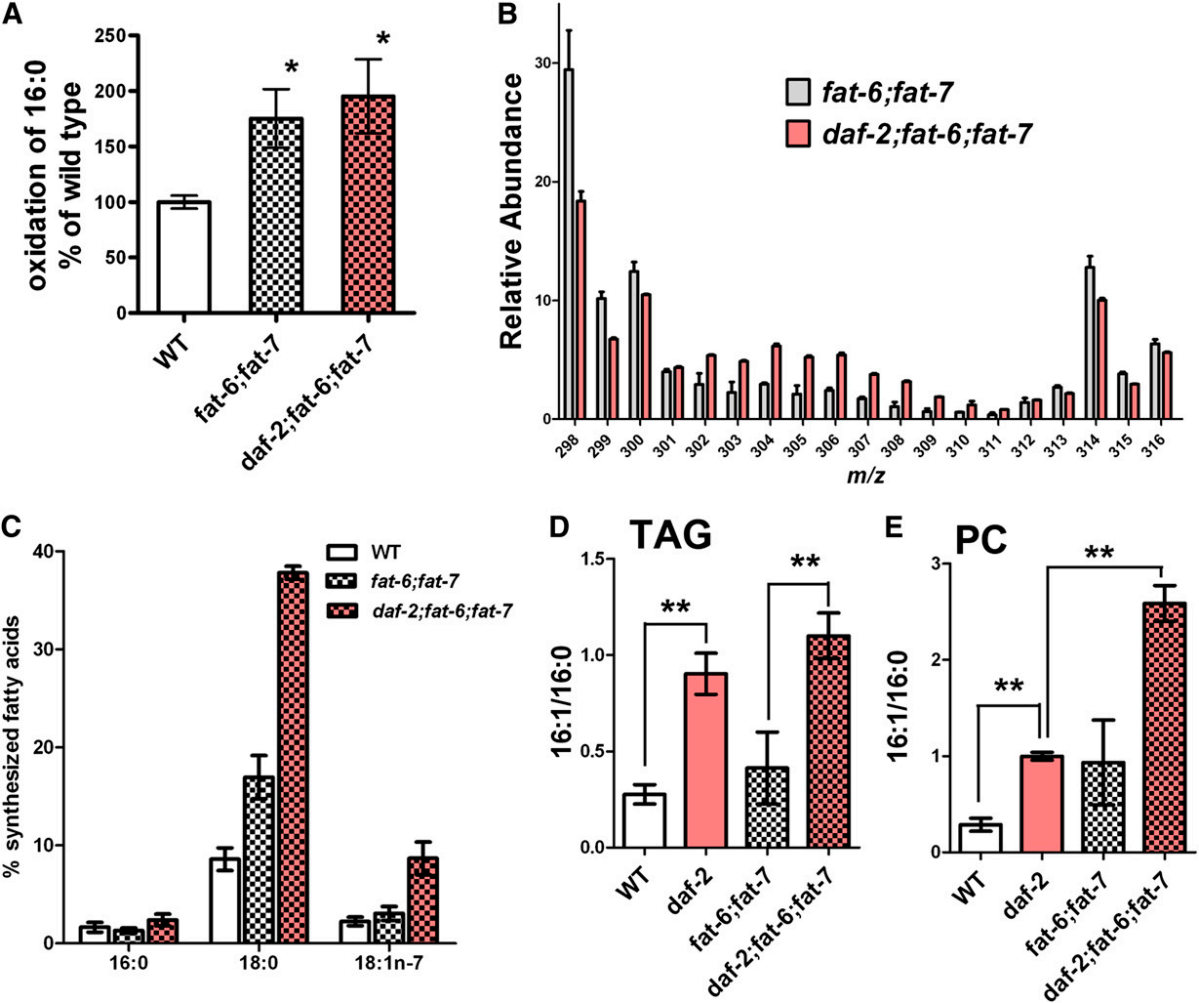
Increased de novo synthesis and FAT-5 activity, not decreased β oxidation, mediates fat storage in the *daf-2* background. (A) Fatty acid oxidation rates are increased in *fat-6;fat-7* and *daf-2;fat-6;fat-7* compared with WT (*P* < 0.05). Nematodes were incubated in 20 μM ^3^H-palmitate (16:0) complexed to BSA, and the amount of tritiated water generated was determined. Values shown are the mean and SEM of 4–6 biological replicates. (B) Isotopomers associated with de novo synthesis (MW302-312) are increased in stearate (18:0) isolated from the total lipids of *daf-2;fat-6;fat-7* (pink) compared with *fat-6;fat-7*. Data are the mean of three experiments (± SD). (C) The percentage of de novo synthesized fatty acids in total lipids is shown for WT, *fat-6;fat-7*, and *daf-2;fat-6;fat-7*. Data are the mean of three experiments (± SEM). (D and E) FAT-5 activity in TAG (D) and PC (E) lipid fractions. Shown is the ratio of the FAT-5 product (16:1) to the FAT-5 substrate (16:0). Error bars are SEM, **P < 0.01, *P < 0.05.

In mice and nematodes, SCD deficiency leads to increased expression of β-oxidation genes, which may lead to increased fat oxidation and reduced fat stores (2, 7). Therefore, we hypothesized that the increased fat stores in the *daf-2;fat-6;fat-7* mutants may be a result of decreased expression of β-oxidation genes in the *daf-2* mutant background. We used real-time quantitative RT-PCR to examine expression of mitochondrial and peroxisomal β-oxidation genes as well as fatty acid-binding protein genes in WT, *fat-6;fat-7*, and *daf-2;fat-6;fat-7* worms. We found that, compared with WT, 12 of 29 genes tested showed increased expression in *fat-6;fat-7* worms. However, the expression remained high in the *daf-2;fat-6;fat-7* worms, indicating that reduced expression of genes encoding β-oxidation machinery is unlikely to be the mechanism for high fat content in *daf-2;fat-6;fat-7* worms (supplementary Table III). Furthermore, a direct assay of fatty acid oxidation activity (30) demonstrated that both the *fat-6;fat-7* and *daf-2;fat-6;fat-7* strains showed higher rates of oxidation of palmitic acid (16:0) than did WT (**Fig. 3A**). We conclude that high TAG accumulation in *daf-2*;*fat-6;fat-7* compared with WT and *fat-6;fat-7* is not a result of decreased fatty acid oxidation.

Previous studies have demonstrated that *daf-2* mutants display increased de novo fatty acid synthesis compared with WT (29). We used stable isotope labeling and GC/MS analysis to determine whether the *daf-2;fat-6;fat-7* triple mutant had increased de novo synthesis compared with the *fat-6;fat-7* double mutant. We found an increased abundance of isotopomers associated with de novo fatty acid synthesis in the *daf-2;fat-6;fat-7* triple-mutant strain compared with *fat-6;fat-7* and WT (Fig. 3B). Interestingly, the *fat-6;fat-7* double mutant showed increased de novo synthesis of stearic acid (18:0), although the relative synthesis of palmitic acid (16:0) and vaccenic acid (18:1n-7) was not different than WT (Fig. 3C). In contrast, the *daf-2;fat-6;fat-7* showed increased de novo synthesis compared with WT and *fat-6;fat-7* in all of the fatty acids analyzed (Fig. 3C). Therefore, mutation in the *daf-2* gene promotes de novo fat synthesis in SCD-deficient worms as well as in WT, and this increased synthesis correlates with increased TAG levels in strains containing the *daf-2* mutation.

The third isoform of Δ9 desaturase in *C. elegans*, FAT-5, is a palmitoyl-CoA desaturase that only is only active on palmitic acid (16:0) and does not desaturate stearic acid (18:0) (6, 43). The *fat-5* gene is upregulated in *fat-6;fat-7* double mutants (7) as well as in *daf-2* mutants (44). To estimate FAT-5 activity, we compared the abundance of the product (16:1) to the precursor (16:0) to estimate the FAT-5 activity. We found that the ratio of palmitoleic acid (16:1) to palmitic acid (16:0) was increased in the *daf-2* and *fat-6;fat-*7 background compared with WT, and strikingly, the 16:1 to 16:0 ratio was greatest in the triple-mutant strain (Fig. 3D, E). The ratio of 16:1 to 16:0 was highest in the *daf-2;fat-6;fat-7* strain in both fat storage lipids (TAG, Fig. 3D) and membrane phospholipids (PC, Fig. 3E). In addition, every lipid class contained higher amounts of PUFAs in the *daf-2;fat-6;fat-7* mutants compared with the *fat-6;fat-7* strain. Because strains containing mutations in both *fat-6* and *fat-7* form unusual PUFAs through the FAT-5 pathway (7), the increased FAT-5 activity is consistent with increased PUFAs in PC as well as other lipid classes in *daf-2;fat-6;fat-7* compared with *fat-6;fat-7* (Fig. 2D, supplementary Table II). This finding suggests that increased FAT-5 activity may facilitate increased TAG synthesis in the *daf-2;fat-6;fat-7* triple mutants.

### SCD deficiency leads to low PC:PE ratio

**Fig. 4.**
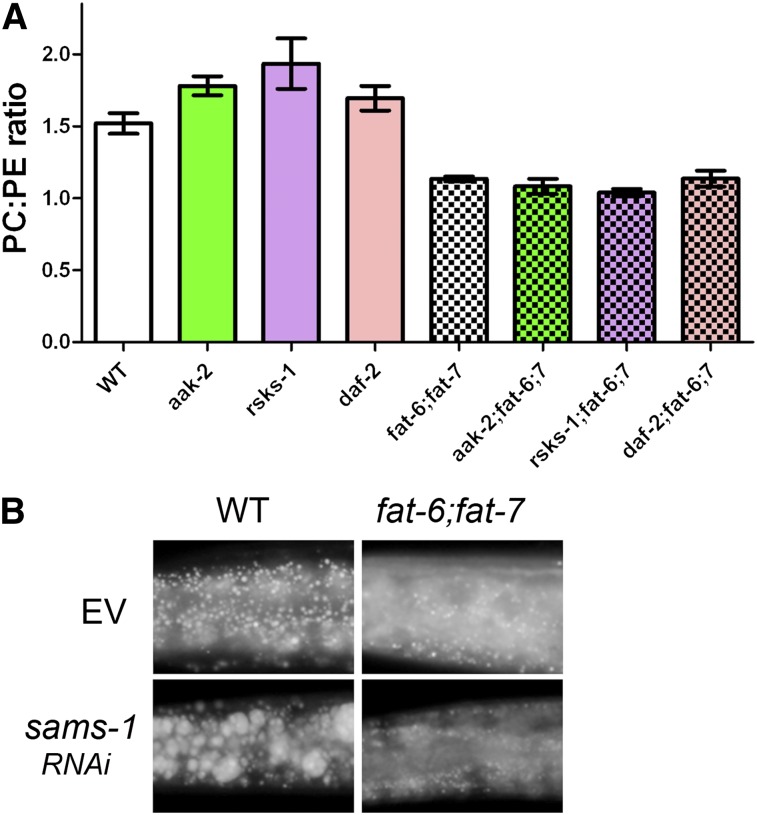
SCD influences PC:PE ratios. (A) The ratio of PC:PE is reduced in strains containing *fat-6;fat-7*. Values are the average PC:PE ratio measured in lipid extracts from three biological replicates of each genotype. (B) RNAi of *sams-1* leads to large-sized lipid droplets in WT, but large-sized droplets do not form in the *fat-6;fat-7* mutant.

Our extensive lipid analysis allowed us to compare the ratios of the two major membrane phospholipids, PC and PE, in all of the strains. The ratio of PC to PE is important for proper membrane function. We found that the ratio of PC:PE among independently grown batches of young adult WT nematodes ranged 1.41–1.65, with an average ratio of 1.51 (**Fig. 4A**, Table 1). The ratio of PC:PE was somewhat higher than WT in the *aak-1*, *rsks-1*, and *daf-2* mutant strains, ranging 1.57–2.26, with an average of 1.78 for *aak-2*, 1.96 for *rsks-1*, and 1.69 for *daf-2* mutants. In contrast, the ratio of PC:PE was less variable and significantly lower in the *fat-6;fat-7* mutant strain, ranging 1.11–1.17, with an average of 1.13. Strikingly, in combination with *aak-2*, *rsks-1*, and *daf-2*, the ratio remained low, ranging 0.99–1.25 in the triple-mutant strains, with an average of 1.08 for *aak-2;fat-6;fat-7*, 1.04 for *rsks-1;fat-6;fat-7*, and 1.13 for *daf-2;fat-6;fat-7* (Fig. 4A, Table 2). This finding indicates that the low PC:PE ratio correlates with the fatty acid composition of the animals, with either the high content of saturated fatty acids or the low content of polyunsaturated fatty acids influencing the relative amounts of PC and PE in membranes.

Reduced amounts of PC are associated with large lipid droplets in *Drosophila* S2 cells (45) and fatty livers in mice (46). Therefore, we were surprised that the small lipid droplets in *fat-6;fat-7* mutants were associated with reduced amounts of PC relative to PE. In *C. elegans*, depletion of *sams-1* leads to diminished PC (47). The *sams-1* gene encodes S-adenosylmethionine synthase, which is required to transfer methyl groups onto PE to form PC, one pathway of PC synthesis. Because the *sams-1* mutants have large lipid droplets (47), consistent with reduced PC content, we used RNAi to deplete *sams-1* in the *fat-6;fat-7* double mutant (Fig. 4B). The large lipid droplets induced by depletion of *sams-1* did not form in the SCD-deficient *fat-6;fat-7* worms, indicating that SCD is essential for lipid droplet expansion, even when PC levels are reduced. Taken together, these studies reveal an important role for SCD activity in the regulation of lipid droplet size, independent of fat accumulation and membrane phospholipid ratios.

## DISCUSSION

**Fig. 5.**
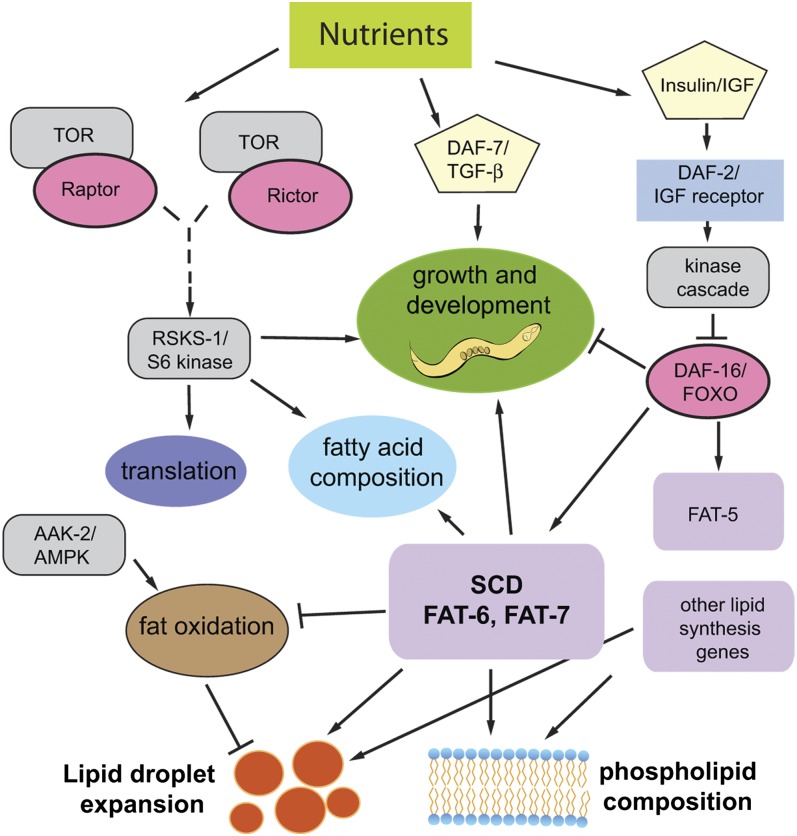
Model and summary depicting the central role of SCD in the regulation of fat stores and development in *C. elegans*. Multiple pathways contribute to proper development and energy allocation. Mutations in the S6 kinase *rsks-1* lead to slow growth, increased fat stores, and altered fatty acid composition. Similarly, mutations genes encoding DAF-7/TGFβ and DAF-2/IIS receptor lead to increased fat stores. Large-sized lipid droplets and high PC:PE ratios in all mutants requires SCD activity, although in *daf-2;fat-6;fat-7* mutants, overall fat stores are improved due to increased activity of the palmitoyl-CoA desaturase FAT-5.

Construction of the *fat-6;fat-7* triple-mutant strains enabled us to ascertain whether various mutations that, on their own, lead to high fat stores in *C. elegans*, are able to overcome the low fat stores of the SCD-defective *fat-6;fat-7* mutants. We found that development and fat storage is affected in unique ways. The combination of TGFβ with SCD in the *daf-7;fat-6;fat-7* strain is lethal, whereas the combination of reduced insulin signaling and SCD in the *daf-2;fat-6;fat-7* triple-mutant strain leads to the ability to store WT levels of fat. In other mutant strains, in which SCD deficiency is combined with mutations in *aak-2*, *sams-1*, and *rsks-1*, growth and fat storage resemble the SCD mutants, revealing an essential role for SCD in efficient fat storage as well as in ensuring proper growth and development. Notably, the FAT-6 and FAT-7 SCDs regulate the size of lipid droplets and ratios of cellular phospholipids in every strain examined. **Fig. 5** depicts a model summarizing the pathways examined in these studies. A link between SCD activity and lipid droplet size was previously observed in cell lines cultured from patients with Berardinelli-Seip congenital lipodystrophy (48). This study showed that patients carrying mutations in the Seipin gene had increased proportions of saturated fatty acid in their lipids, indicating decreased SCD activity and decreased size and abundance of lipid droplets.

Our extensive lipid analysis revealed previously unknown lipid composition defects in the S6kinase-deficient *rsks-1* strain. We found that the overall TAG levels were high in this strain, consistent with larger lipid droplets observed by Nile Red staining of fixed worms. We also identified increased omega-6 and decreased omega-3 polyunsaturated fatty acids in the membrane lipid components (supplementary Fig. I, supplementary Table II). Although upstream components of the TOR signaling pathway, such as *daf-15*/RAPTOR, *rict-1*/RICTOR, and *let-363*/TOR, also have increased fat stores (15, 19), we did not identify the alteration in the membrane omega-3:omega-6 ratios in *rict-1* mutants, although we detected similar fatty acid composition changes in ribosomal protein RNAi knockdown worms (supplementary Fig. II). Therefore, the *rsks-1* mutants and knockdowns in proteins required for translation have an altered fatty acid composition that is not observed in *rict-1* mutants.

In spite of the decreased lipid droplet size, the abundance of lipid droplets, as well as the total TAG levels in the worms, was increased in the *daf-2* mutant background. This was not due to a decrease of β-oxidation conferred by the *daf-2* mutation, because we found that fatty acid oxidation rates were increased in both the *fat-6;fat-7* and *daf-2;fat-6;fat-7* strains (Fig. 3). Instead, increased de novo synthesis correlates with the increased fat stores in strains containing the *daf-2* mutation (29). Our findings indicate that the induction of the FAT-5 palmitoyl-CoA desaturase facilitates the increase in de novo fat synthesis.

Mice lacking SCD1 are lean and resistant to diet-induced obesity (2, 49). They have reduced fatty acid and TAG synthesis in response to high dietary carbohydrates (50), and they also have increased fat oxidation rates in various tissues (49). Cell culture studies of lipid droplet formation rely on oleic acid in the culture media to induce the expansion of lipid droplets (45). However, our studies in an intact organism reveal that dietary oleic acid does not fully compensate for the endogenous synthesis of monounsaturated fatty acids. This is consistent with mouse studies that revealed that diets containing up to 5% oleate did not rescue the low fat stores in SCD1 mutants (50) and with human studies that showed that diets high in oleic acid, such as a Mediterranean diet, provide a protective role against obesity, whereas diets high in saturated fats and simple carbohydrates, which induce endogenous SCD activity, lead to excess fat stores (51).

Interestingly, the SCD1 was shown to colocalize with diacylglycerol acyltransferase (DGAT)2 in the mitochondrial-associated membrane subcompartment of the endoplasmic reticulum (ER) (52). DGAT enzymes are required for the formation of lipid droplets (53). The colocalization of SCD1 and DGAT2 suggests that metabolic channeling of endogenously synthesized monounsaturated fatty acids and DGAT2 optimizes TAG synthesis. Work in *C. elegans* demonstrated that DGAT2 and ACS-22 form a complex at the ER-lipid droplet interface during lipid droplet expansion (54). Although we neither observed FAT-6::GFP or FAT-7::GFP on the lipid droplet surface nor were these gene products identified in a proteomic analysis of *C. elegans* lipid droplets (25), we found that endogenous SCD activity is necessary for efficient lipid storage in *C. elegans*, and we found that dietary oleic acid did not restore large-sized lipid droplets, consistent with the metabolic channeling hypothesis. A recent study provides evidence that lipogenic enzymes, specifically GPAT4, relocalize from the ER to a subset of expanding lipid droplets (55). It is possible that proper membrane composition may be required for efficient relocalization of TAG synthesis enzymes to expanding lipid droplets.

In mice, *Drosophila*, and *C. elegans*, disruption of PC synthesis leads to increased TAG and large-sized lipid droplets (45–47, 56). Upon lipid loading of cells, PC is synthesized and its presence prevents the coalescence of lipid droplets (45). Our studies show that all of the SCD-deficient strains have a decreased amount of PC relative to PE. This data is in agreement with a recent metabolic study of *C. elegans* SCD mutants which revealed a reduction in some phosphocholine derivatives in SCD mutants compared with WT (57). The small-sized lipid droplets in the *fat-6;fat-7* double mutants seem contradictory to the reduced PC, since reducing PC synthesis leads to large lipid droplets. Even so, we show that SCD activity is required for the large-sized droplets in the PC-deficient *sams-1* mutants. Therefore, even though PC levels are reduced in *fat-6;fat-7* mutants, the levels are apparently adequate to prevent the coalescence of lipid droplets. Instead, the lipid droplet size appears to be driven by the ability of SCD to synthesize unsaturated fatty acids. It is tempting to speculate that the alteration in the PC:PE ratio represents a compensation for the increased presence of saturated fatty acids and decreased abundance of PUFAs in the SCD-deficient strains. For example, PC molecules containing unsaturated fatty acid are typically cylinder shaped, whereas PE molecules, with a smaller headgroup, are typically cone shaped (58). Because the chain length and the relative degree of unsaturation influences the shape of the fatty acids, which in turn influences the phospholipid shape, we predict that the shape of the PC molecules may be altered in *fat-6;fat-7* double mutants. The decrease in PC together with the increase in PE may provide a phospholipid composition that improves membrane function in the context of increased saturated fatty acids in the *fat-6;fat-7* strains.

## Supplementary Material

Supplemental Data
