# Supplementary material for: Regulation of lipid droplet size and phospholipid composition by stearoyl-CoA desaturase
Source: J Lipid Res. 2013 Sep;54(9):2504–14. doi: 10.1194/jlr.M039669 (PMC3735947; doi:10.1194/jlr.M039669)
Supplement: Supplemental Data [file supp_54_9_2504__index.html]

The regulation of lipid droplet size and phospholipid composition by stearoyl-CoA desaturase — Regulation of lipid droplet size and phospholipid composition by stearoyl-CoA desaturase — Supplemental Data 

# Regulation of lipid droplet size and phospholipid composition by stearoyl-CoA desaturase

## 

**Files in this Data Supplement:**

- Supplemental Figure 1 - Figure S1. A. Severe SCD deficiency reduces lipid droplet size in C. elegans. Mutations in fat-5,fat-6, or fat-7 or the double mutation fat-5;fat-6 do not visibly reduce lipid droplet size wild type or in daf-2. The most severe reduction in lipid droplet size in wild type and daf-2 occurs in the fat-6;fat-7 double mutant strain. B. Supplementation of fat-6;fat-7with 0.1mM &#x26;ndash; 0.5mM sodium oleate leads to larger lipid droplets in fat-6;fat-7 worms, but does not completely rescue the lipid droplet size to that of wild type.
- Supplemental Figure 2 - Figure S2. A. Lipid droplet size in wild type, aak-2 or aak-2;fat-6;fat-7 strains is not affected by treatment with aak-1(RNAi). B. Mutation in rict-1 does not increase lipid droplet size in fat-6;fat-7 double mutants. C. The rict-1 and rsks-1 mutant strains develop slower than wild type, and exacerbate the slow growth in the fat-6;fat-7 background. The graph shows the percentage of a population that reached adulthood by the indicated time (0 hours = 1-32 cell stage embryo). D-E. Partial fatty acid composition of rict-1(D), rsks-1(E), and rsps-9(RNAi)(F). Graphs show the relative amounts of C20PUFAs in total lipids (D) or in the phosphatidylcholine (PC) fraction (E and F).
- Supplemental Table 1 - Table S1. Fatty acid composition of lipid classes in wild type (N2) and fat-6;fat-7 double mutants, grown without fatty acids and supplemented with 0.2mM sodium oleate (18:1n-9).
- Supplemental Table 2 - Table S2. Fatty acid composition of lipid classes in wild type (N2), aak-2, rsks-1, daf-2, fat-6;fat-7, aak-2;fat-6;fat-7, rsks-1;fat-6;fat-7, and daf-2;fat-6;fat-7.
- Supplemental Table 3 - Table S3. Changes in gene expression in L4 stage fat-6;fat-7 and daf-2; fat-6;fat-7 compared to wild type.
