# Supplementary figures and images for: Regulation of lipid droplet size and phospholipid composition by stearoyl-CoA desaturase
Source: J Lipid Res. 2013 Sep;54(9):2504–14. doi: 10.1194/jlr.M039669 (PMC3735947; doi:10.1194/jlr.M039669)

**A**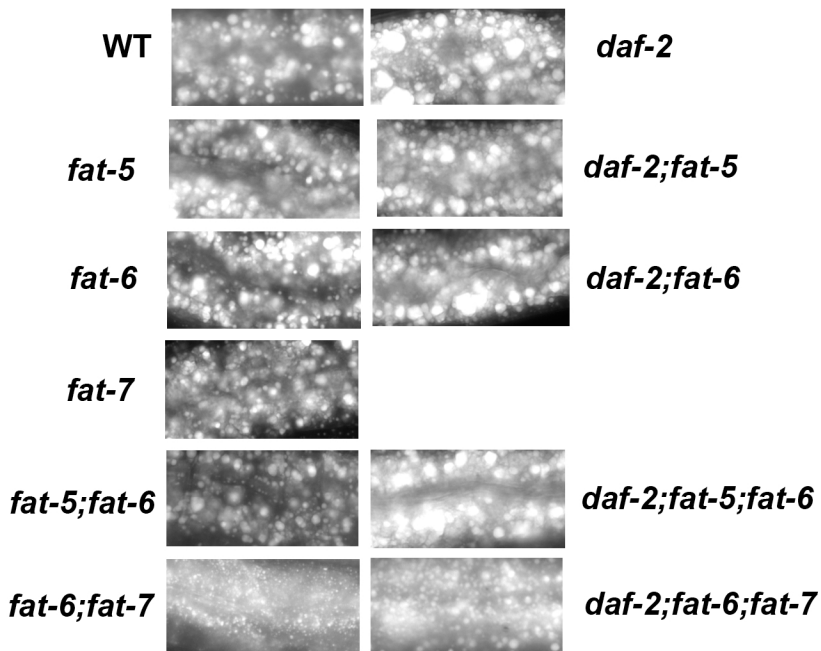**B**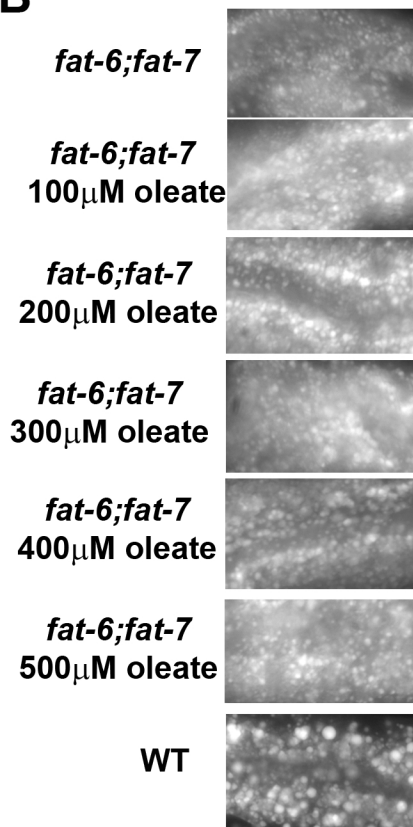

Supplement: Supplemental Data [file supp_M039669_jlr.M039669-1.pdf]

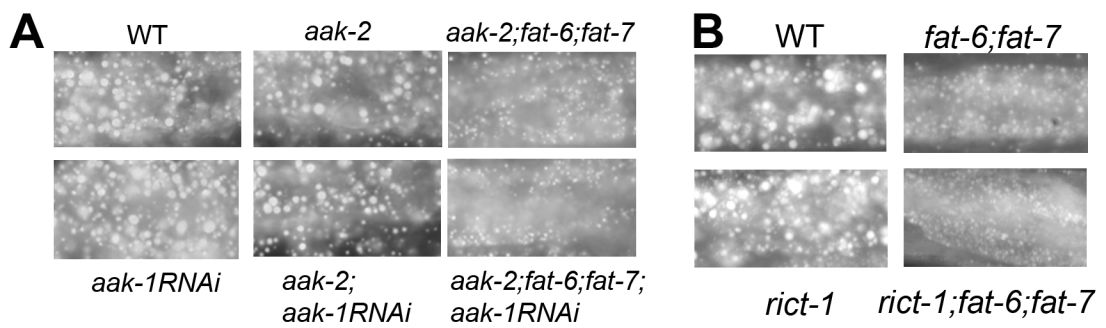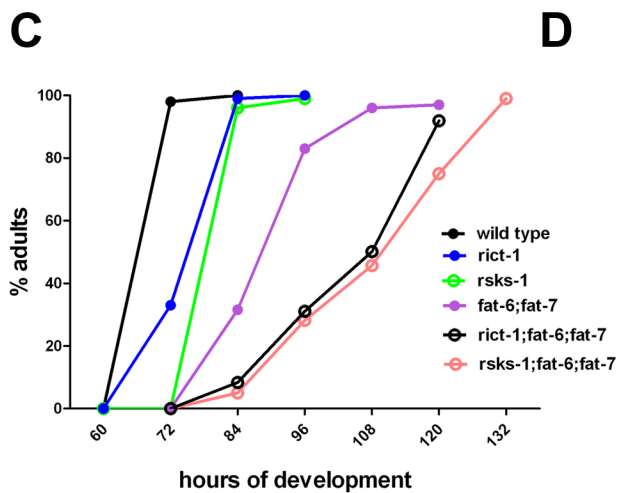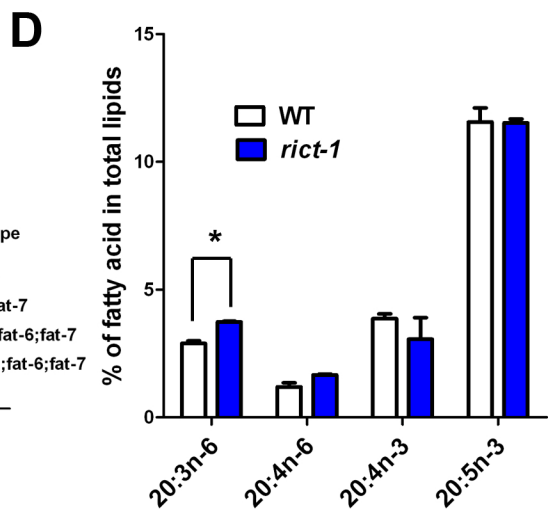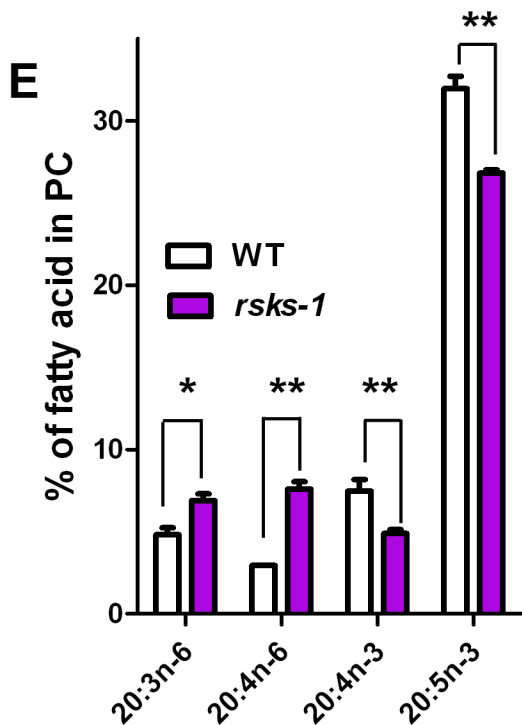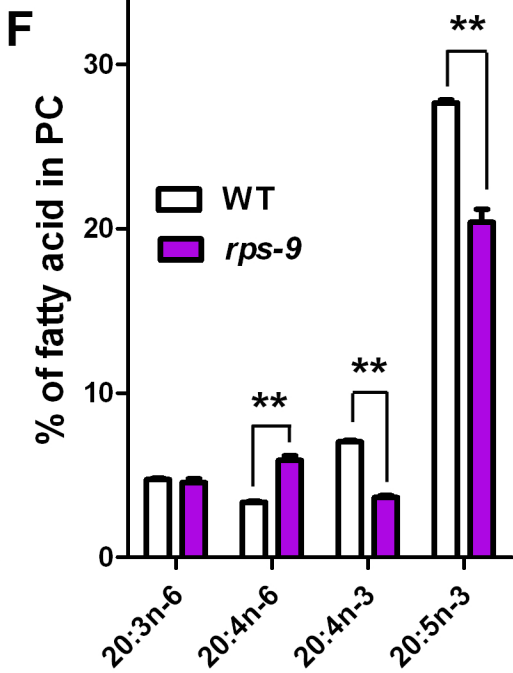

Supplement: Supplemental Data [file supp_M039669_jlr.M039669-2.pdf]
